# Structural equation modeling of the associations between amygdala activation, personality, and internalizing, externalizing symptoms of psychopathology

**DOI:** 10.1017/pen.2020.8

**Published:** 2020-07-14

**Authors:** Craig S. Neumann

**Affiliations:** Department of Psychology, University of North Texas, Denton, TX, USA

**Keywords:** Five-factor model, Personality traits, Amygdala activation, Externalizing and internalizing psychopathology, Structural equation modeling (SEM)

## Abstract

There is an expanding literature on the theoretical and empirical connections between personality and psychopathology, and their shared neurobiological correlates. Recent cybernetic theories of personality and psychopathology, as well as affective neuroscience theory, provide grounding for understanding neurobiological–personality–psychopathology (NPP) associations. With the emergence of large sample datasets (e.g., Human Connectome Project) advanced quantitative modeling can be used to rigorously test dynamic statistical representations of NPP connections. Also, research suggests that these connections are influenced by sex, and large samples provide the opportunity to examine how NPP associations might be moderated by sex. The current study used a large sample from the Duke Neurogenetics Study (DNS) to examine how amygdala activation to facial expressions was linked with self-report of personality traits and clinical interviews of internalizing and externalizing symptoms of psychopathology. Structural equation modeling results revealed direct associations of amygdala activation with personality trait expression, as well as indirect associations (though personality) with symptoms of psychopathology. Moreover, the NPP links were moderated by sex. The current results are in line with research that identifies a broader role played by the amygdala in personality and provide potential insights for continued research in personality neuroscience and recent theories on the neurobiology of personality.

Research has documented a personality–psychopathology connection (Kotov et al., 2010; Krueger & Tackett, 2003). With empirical articulation of the structure of psychopathology (Wright et al., 2013), it is evident that higher order (Big Five) domains of personality (and personality pathology) can be understood with reference to dimensions of psychopathology, such as internalizing and externalizing (Kotov et al., 2011; Widiger et al., 2018). Also, given that personality involves a “dynamic organization within the individual of those *psychophysical* systems that determine … characteristic behavior and thought” (Allport, 1961, p. 28, emphasis added), it is not surprising that neurobiological systems involved in internalizing and externalizing (Goodkind et al., 2015) are also pertinent to personality (DeYoung, 2010a, b). Thus, shared aspects of neurobiology may account for the personality–psychopathology association (DeYoung & Krueger, 2018a; Hyatt et al., 2019). The amygdala is now understood as playing a broader role than originally thought (Cunningham & Brosch, 2012; Pessoa, 2010), and may be a critical area for understanding neurobiological–personality–psychopathology (NPP) links.

Gray’s (1982) research elucidated two broad biological systems – the behavioral inhibition (BIS) and behavioral activation (BAS) systems – which focused attention on neurobiological aspects of personality (Corr & Cooper, 2016; Hoppenbrouwers et al., 2015) and psychopathology (Johnson et al., 2003). Subsequent research revealed the role of the amygdala in the BIS (Barrós-Loscertales et al., 2006) and BAS (Passamonti et al., 2008), and thus implicated the amygdala in NPP associations. Moreover, it has become evident that the amygdala is involved in far more than fear conditioning as initially conceived (Pessoa, 2010). Specifically, the amygdala has been linked to general personality (e.g., Aghajani et al., 2014; Canli et al., 2002; Cunningham et al., 2010; DeYoung & Gray, 2009; DeYoung & Krueger, 2018a) and pathological personality (e.g., Baskin-Sommers et al., 2016; Carré et al., 2013; Donegan et al., 2003; Schultz et al., 2016), as well as psychological processes closely related to personality and psychopathology, including emotional processing (Pessoa & Adolphs, 2010), social cognition (Adolphs, 2010), and self-awareness and self-regulation (Christoff et al., 2011; Northoff et al., 2006; Vago & David, 2012). More generally, the amygdala appears to play a role in ‘tuning’ motivational significance and goals (Cunningham & Brosch, 2012). Similarly, the amygdala has been described as playing a fundamental role in attention, value representation, and decision-making (Pessoa, 2010). Based on extensive animal research and a more ‘bottom-up’ approach, investigators have proposed that subcortical systems (including the amygdala) are foundational for personality development in both humans and animals (Davis & Panksepp, 2011; Latzman et al., 2018).

That amygdala activation is linked to values and motivational aspects of organisms, as well as personality development, highlights its relevance to expression of personality and psychopathology (Davidson, 2000; Davidson et al., 2000). For instance, studies have indicated that a range of characteristics of people is linked with amygdala *activation*, such as Neuroticism and depression (Hariri, 2009), trait optimism (Feder et al., 2009), trait happiness and positive emotion (Cunningham & Kirkland, 2014), attachment style (Vrticka et al., 2008), resilience (Heitzeg et al., 2008), pathological personality traits (Carré et al., 2013; Donegan et al., 2003; Schultz et al., 2016), and personality style and attentional focus (Most et al., 2006). In addition, recent research suggests considerable neuroanatomical overlap (including the amygdala) between personality traits and internalizing, externalizing psychopathology (Hyatt et al., 2019), consistent with the notion of fundamental NPP links. An important consideration is how the amygdala might fit into a larger theory regarding such links.

DeYoung and colleagues (DeYoung 2010a; DeYoung & Gray, 2009) have been actively pursuing trait–brain associations, and proposed a Cybernetic Big Five Theory (CB5T; DeYoung, 2015) and a Cybernetic Theory of Psychopathology (CTP; DeYoung & Krueger, 2018a) that offer explanatory, causal theories of personality and psychopathology in terms of neurobiology. These theories posit personality and psychopathology reflect a cybernetic system involved in goal pursuit, with traits viewed as probabilistic descriptions of behavior influenced by neurobiological systems (e.g., serotonin, dopamine, frontal, subcortical). Essential elements of cybernetic systems are stability of goal pursuit and flexibility to adapt as needed to continue to pursue initial or new goals. Psychopathology results when there is “persistent failure to move toward one’s goals due to failure to generate effective new goals, interpretations, or strategies when existing ones prove unsuccessful” (DeYoung & Krueger, 2018a, p. 121).

The Big Five domains can be specified in terms of two hierarchical meta-traits – stability (Emotional Stability or low Neuroticism, Agreeableness, and Conscientiousness) and plasticity (Extroversion, Openness) – which represent crucial aspects of a cybernetic personality system. As DeYoung (2015) states, stability and plasticity are “complementary and, also, in dynamic tension, as extreme plasticity may pose a challenge to stability and vice versa. The opposite of stability is not plasticity but instability, and the opposite of plasticity is not stability but rigidity” (p. 47). That CB5T involves a neurobiological system linked to goals and motivations, and the amygdala is now discussed in terms of values and motivational goal significance, suggests there should be meaningful links between amygdala activity, personality trait expression, and emergence of symptoms of psychopathology. As a social species, human motivational goals for affiliation play a prominent role in personality trait expression (DeYoung & Krueger, 2018a; Neumann et al., 2020), and to the extent there is cybernetic dysfunction of affiliative motives (psychological entropy beyond the edge of chaos, DeYoung & Krueger, 2018b), risk for psychopathology increases (Viding & McCrory, 2019).

In terms of specific NPP links, Neuroticism, which is inversely associated with affiliative traits (Jang et al., 2001), is regularly discussed with respect to increased amygdala activation and depression (Hariri, 2009), though it appears that different aspects of Neuroticism may modulate these associations (Cunningham et al., 2010), as well as serotonin polymorphism (Hariri et al., 2002; Murphy et al., 2013), or presence of stressful conditions (Everaerd et al., 2015). Conversely, Extroversion (E), intimately linked with sociability, is robustly associated with the amygdala activation (Aghajani et al., 2014; Canli et al., 2002). That Neuroticism and Extroversion are both positively associated with amygdala activity suggests activation is not necessarily problematic (Cunningham & Kirkland, 2014), particularly if it is associated with adaptive goals, motivations, and values (Cunningham & Brosch, 2012; Pessoa, 2010). Important considerations regarding personality is how it may play a role in the regulation of amygdala activity (Gresham & Gullone, 2012; Hermann et al., 2014; Matsumoto, 2006; Morawetz et al., 2017), and of course, that the amygdala is part of larger neural systems (e.g., Bickart et al., 2012; Drabant et al., 2009; Ousdal et al., 2012), which have implications for NPP links (Hermann et al., 2014; Holmes et al., 2012).

In this context, it is noteworthy that, in addition to Neuroticism, the two other stability traits, Agreeableness and Conscientiousness, have links to the serotonin system (DeYoung & Krueger, 2018a). Disturbances in this system are associated with amygdala activation (Murphy et al., 2013; Volman et al., 2013), though effective emotion regulation is associated with the downregulation of amygdala activity (Drabant et al., 2009; Hölzel et al., 2013; Joormann et al., 2012; Monk et al., 2008). As it turns out, Agreeableness appears to be associated with emotion regulation, via the lateral prefrontal cortex (Haas et al., 2007), which is involved in an amygdala-cortical circuit that down-regulates amygdala activity (Drabant et al., 2009; Hermann et al., 2014; Modinos et al., 2010). Relatedly, aspects of social affiliation, conceptually related to Agreeableness, are also associated with amygdala–cortical connectivity (Bickart et al., 2012). Taken together, the pattern of findings suggest that amygdala activation should be inversely related to Agreeableness.

The connection between Conscientiousness and the amygdala is less straightforward. Conscientiousness has been found to be associated with the prefrontal cortex (DeYoung & Gray, 2009) and the default mode network (Beaty et al., 2016; Toschi et al., 2018). Yet, the DMN (Jiang et al., 2016) and PFC (Modinos et al., 2010) have links with the amygdala. Also notable is that psychopathic and borderline personality are both associated with emotion dysregulation (Garofalo, Neumann, & Mark, 2020; Wupperman et al., 2009 ), amygdala hyperactivity (Carré et al., 2013; Donegan et al., 2003; Schultz et al., 2016), as well as low Agreeableness and Conscientiousness (Distel et al., 2009; Seara-Cardoso et al., 2020). Thus, it seems reasonable to suggest that Conscientiousness may also be inversely linked with amygdala activity.

Finally, Openness, perhaps the least understood with respect to neurobiology, appears to have links to higher level cognitive abilities, such as attentional control, which are associated with the PFC (DeYoung & Gray, 2009). Recent research has further supported the link between Openness and cognition (DeYoung, 2014). To the extent that the PFC plays a role in amygdala down-regulation, one might suspect an inverse association between Openness and amygdala activation. Consistent with this idea, Openness and regular use of reappraisal are associated with successful ability to down-regulate amygdala activity (Morawetz et al., 2017). Also, reappraisal is linked with Openness (Gresham & Gullone, 2012). One the other hand, Openness and Extroversion are the two plasticity traits, and the latter trait has been linked with amygdala activation. Thus, it is not too surprising perhaps that Morawetz and colleagues found that aspects of Openness (identify feelings) were associated with the ability to up-regulate amygdala activity (Morawetz et al., 2017). Taken together, it is difficult to propose how Openness might be related to amygdala activity, though it appears that it may represent one of the more dynamic traits with respect to NPP associations.

How might sex influence NPP associations? Unfortunately, various studies often control for sex (e.g., Gray et al., 2018; Moore et al., 2018; Yang et al., 2020) or combine males and females in a single sample without any control for sex (e.g., Scheffel et al., 2018). These may be unfortunate choices, given that sex differences have been documented in terms of amygdala activation (Stevens & Hamman, 2012), personality traits (Schmitt et al., 2008), and internalizing, externalizing psychopathology (Hasin & Grant, 2015). Why might sex differences be relevant? Males and females also differ in emotion regulation and corresponding neurobiological activity (Domes et al., 2010). With respect to personality and psychopathology, DeYoung et al. (2008) found that decreases in stability and increases in plasticity traits predicted externalizing psychopathology in a male sample. However, large sample behavior genetic research finds a common genetic basis for externalizing and Extroversion in only women, and with Sensation Seeking (i.e., low Conscientiousness) only in men, while Novelty Seeking had a similar genetic basis with externalizing in both males and females (Kendler & Myers, 2014). Taken together, the pattern of results suggests that the link between neurobiology, personality, and externalizing may be influenced by sex. At this point, it is difficult to generate specific a priori hypotheses on how sex may moderate NPP links, but, nonetheless, it is fair to suggest that sex should moderate them given sex differences among the three NPP domains.

The current study relied on a large sample of archival data from the Duke Neurogenetics Study (Elliot et al., 2018; Prather et al., 2013; Swartz et al., 2017) with access to functional brain imaging data of the amygdala, Big Five traits, and symptoms of internalizing (INT), and externalizing (EXT) psychopathology. The functional imaging data drew on a facial expression (fearful, angry, surprised) paradigm that been shown to evoke robust (Nickolov et al., 2016; Prather, Bogdan, & Hariri, 2013) and reliable (Manuck, Brown, Forbes, & Hariri, 2007) amygdala reactivity. Examination of amygdala activation to facial stimuli is advantageous to the extent that it taps into interpersonal processes (e.g., reading emotion in a person’s face) and thus has relevance for CB5T. Specifically, in CB5T, “personality traits are probabilistic descriptions of relatively stable patterns of emotion, motivation, cognition, and behavior, in response to *classes of stimuli* that have been present in human environments over evolutionary time” (DeYoung & Krueger, 2018a, emphasis added, p. 122). Examples of such stimuli are threats, rewards, or other people. Human faces represent a powerful person stimulus that activates the amygdala (Hariri, 2009), and cortico-amygdala connectivity has been shown to be significantly associated with increased affiliation (Bickart et al., 2012), and affiliation represents a cybernetic psychological goal that is fundamentally tied to both traits and brain mechanisms (DeYoung & Krueger, 2018a).

Based on the review of the literature, several NPP associations were expected to be uncovered in the present study. Since serotonin is associated with stability traits (DeYoung & Krueger, 2018a) and problems with this neurotransmitter system are associated with amygdala activation (Hariri et al., 2002; Murphy et al., 2013), one would expect that activation should be linked to the Stability traits. With respect to Neuroticism, this is a difficult prediction, given variations in the form of neuroticism (Cunningham et al., 2010), genetic polymorphisms (Hariri et al., 2002; Murphy et al., 2013), and experimental conditions (Everaerd et al., 2015) affect the link with amygdala activation. On the other hand, Agreeableness (Haas et al., 2007) and Conscientiousness (DeYoung & Gray, 2009) both have links to neurobiological systems involved in emotion regulation (Drabant et al., 2009; Hermann et al., 2014; Modinos et al., 2010), which is associated with the down-regulation of amygdala activity (Morawetz et al., 2017). As such, one would expect decreased activation of the amygdala should be associated with increased Agreeableness and Conscientiousness. The association between Extroversion and positive emotion is well established (DeYoung, 2015), and extroversion, as well as trait optimism and happiness, is associated with amygdala activation (Aghajani et al., 2014; Canli et al., 2002; Cunningham & Kirkland, 2014; Feder et al., 2009). Thus, amygdala activation should be associated with increased Extroversion in the present study. At present, no predictions could be made for Openness and amygdala activation. On the other hand, based on a wealth of literature (Kotov et al., 2010), it was expected that the personality trait scales would have uniform associations with INT and EXT across the males and females, particularly a positive association between Neuroticism and INT, as well as negative associations between Agreeableness, Conscientiousness, and EXT. Extroversion was also expected to be positively associated with EXT (DeYoung et al., 2008; Ruiz et al., 2008), especially for females (Kendler & Myers, 2014).

Finally, how to model the personality–psychopathology link remains an open area. DeYoung and Krueger (2018b) stated that “signs and symptoms of psychopathology have proven empirically to be on the same latent continua as personality traits. Thus, it is valuable to understand the neural variables that underlie these continua” (p. 166). While research has found meaningful empirical associations between personality and psychopathology, at present it is often the case that each domain is viewed as separate but inter-correlated. For instance, antagonism (or low Agreeableness) and low Conscientiousness are strongly related to but not modeled or empirically aggregated as synonymous with EXT (DeYoung et al., 2008; Kotov et al., 2011; Ruiz et al., 2008), and a similar case can be made for neuroticism and INT (Klein et al., 2011; South & Krueger, 2011). Thus, two models were tested which represented the personality and psychopathology domains as separate (Model A) versus specifying both domains as indicators of broader INT and EXT latent variables (Model B).

## 1. Methods

### 1.1 Participants

A large sample of young adults (*N* = 1,330, females = 762) was assessed for Big Five traits, symptoms of psychopathology, and participated in a robust and active imaging lab (Duke Neurogenetics Study; Romer et al., 2018). The DNS was approved by the Duke University School of Medicine Institutional Review Board, and all participants provided written informed consent prior to participation. Study exclusions included 1) medical diagnoses of cancer, stroke, diabetes requiring insulin treatment, chronic kidney or liver disease, or lifetime history of psychotic symptoms; 2) use of psychotropic, glucocorticoid, or hypolipidemic medication; 3) conditions affecting cerebral blood flow and metabolism (e.g., hypertension). Current and lifetime DSM-IV (the Diagnostic and Statistical Manual of Mental Disorders) Axis I or select Axis II disorders were assessed with the electronic Mini International Neuropsychiatric Interview (Lecrubier et al., 1997) and Structured Clinical Interview for the DSM-IV Axis II subtests (First et al., 1997), respectively. Neither current nor lifetime diagnosis was an exclusion criterion, given the DNS goal to understand the broad variability in multiple behavioral phenotypes related to psychopathology. Nevertheless, no participant, regardless of diagnosis, was taking any psychoactive medication during or at least 14 days prior to their participation.

### 1.2 Personality and psychopathology symptom measures

The 240-item NEO personality inventory revised (NEO-PI-R; Costa and McCrae, 1995) was used to assess Big Five personality domains: 1) Neuroticism (N), 2) Agreeableness (A), 3) Conscientiousness (C), 4) Extraversion (E), and 5) Openness-to-Experience (O). All domains are composed of six facets each with eight items. Each Big Five personality domain was a sum of the facet scores (accounting for reverse coded items). NEO items are on a scale ranging from (0) strongly disagree to (4) strongly agree. Internal consistency of the personality traits was assessed by Cronbach’s alpha as fair to good, ranging between .70 and .85. Consistent with previous research (Schmitt et al., 2008), females showed slightly higher scores on the NEO domains, though the effect sizes were quite small (*η* ^*2*^ range = .002 [Openness] to .02 [Neuroticism]).

Based on previous DNS research (Romer et al., 2018), symptoms of Internalizing (INT) and Externalizing (EXT) were also available. These variables were obtained via the electronic Mini International Neuropsychiatric Interview and multiple self-report mental health questionnaires which assessed 1) internalizing symptoms of depression, generalized anxiety, and fears/phobias; 2) externalizing symptoms of antisocial personality/psychopathy, delinquency, and alcohol, cannabis, and other drug abuse/dependence. The INT and EXT variables were standardized to a mean of 100 (SD = 15), with higher scores indicating a greater propensity for psychiatric symptoms (Romer et al., 2018). Consistent with previous research (Hasin & Grant, 2015), females showed slightly higher INT scores and males higher EXT scores, though the effects sizes were small (*η* ^*2*^’s = .01 [INT] and .06 [EXT]).

### 1.3 Functional MRI data

#### 1.3.1 BOLD fMRI data acquisition

Each participant was scanned using a research-dedicated GE MR750 3 T scanner equipped with high-power high-duty cycle 50-mT/m gradients at 200 T/m/s slew rate, and an eight-channel head coil for parallel imaging at high bandwidth up to 1MHz at the Duke-UNC Brain Imaging and Analysis Center. A semi-automated high-order shimming program was used to ensure global field homogeneity. A series of 34 interleaved axial functional slices aligned with the anterior commissure–posterior commissure plane were acquired for full-brain coverage using an inverse-spiral pulse sequence to reduce susceptibility artifacts (TR/TE/flip angle = 2000 ms/30 ms/60; FOV = 240 mm; 3.75 × 3.75 × 4 mm voxels; interslice skip = 0). Four initial radiofrequency excitations were performed (and discarded) to achieve steady-state equilibrium. To allow for spatial registration of each participant’s data to a standard coordinate system, high-resolution three-dimensional structural images were acquired in 34 axial slices coplanar with the functional scans (TR/TE/flip angle = 7.7 s/3.0 ms/12; voxel size = 0.9 × 0.9 × 4 mm; FOV = 240 mm, interslice skip = 0). Further discussion of DNS methods can be found at the following website: https://www.haririlab.com/methods/amygdala.html.

#### 1.3.2 BOLD fMRI data pre-processing

Anatomical images for each subject were skull stripped, intensity normalized, and nonlinearly warped to a study-specific average template in a standard stereotactic space (Montreal Neurological Institute template) using ANTs (Klein & Tourville, 2012). BOLD time series for each subject were processed in AFNI (Cox, 1996). Images for each subject were despiked, slice-time corrected, realigned to the first volume in the time series to correct for head motion, coregistered to the anatomical image using FSL’s Boundary Based Registration (Greve & Fischl, 2009), spatially normalized into MNI space using the non-linear warp from the anatomical image, resampled to 2-mm isotropic voxels, and smoothed to minimize noise and residual difference in gyral anatomy with a Gaussian filter, set at 6 mm full-width at half-maximum. All transformations were concatenated so that a single interpolation was performed. Voxel-wise signal intensities were scaled to yield a time series mean of 100 for each voxel. Volumes exceeding 0.5-mm frame-wise displacement or 2.5 standardized DVARS (Nichols, 2017; Power et al., 2014) were censored from the GLM.

#### 1.3.3 fMRI quality assurance criteria

Quality control criteria for inclusion of a participant’s imaging data were >5 volumes for each condition of interest retained after censoring for FD and DVARS and sufficient temporal SNR within the bilateral amygdala, defined as greater than 3 standard deviations below the mean of this value across subjects. The amygdala was defined using a high-resolution template generated from 168 Human Connectome Project datasets (Tyszka & Pauli, 2016). Additionally, data were only included in further analyses if the participant demonstrated sufficient engagement with the task, defined as achieving at least 75% accuracy during the face matching condition.

#### 1.3.4 fMRI paradigm

Participants took part in a fMRI paradigm designed to elicit amygdala responses which involved a face-matching paradigm that has been shown to evoke robust (Prather, Bogdan, & Hariri, 2013) and reliable (Manuck, Brown, Forbes, & Hariri, 2007) amygdala reactivity across a wide range of populations. This task has been described in detail in previously published research from the Duke Neurogenetics Study (Prather et al., 2013; Swartz et al., 2017) and has been used extensively to elicit amygdala activity across an array of experimental protocols and sample populations. Also, the activation task was successfully employed to link personality traits to amygdala responses (Carré et al., 2013).

The task consists of four experimental blocks interleaved with five control blocks. In the DNS version of this task, there is one experimental block each of fearful, angry, surprised, and neutral facial expressions presented in a pseudorandom order across participants. During these experimental blocks, participants view a trio of faces and select one of two faces (on the bottom) identical to a target face (top level). Each of these blocks consists of six images, balanced for gender, all of which were derived from a standard set of pictures of facial affect (Ekman & Friesen, 1976). During the control blocks, participants view a trio of simple geometric shapes (circles and vertical and horizontal ellipses) and select one of two shapes (bottom) that are identical to a target shape (top). Each of these blocks consists of six different shape trios. All the blocks are preceded by a brief instruction (“Match Faces” or “Match Shapes”) that lasts 2 s. In the experimental task blocks, each of the six face trios is presented for 4 s with a variable interstimulus interval (ISI) of 2–6 s (mean = 4 s) for a total block length of 48 s. A variable ISI is used to minimize expectancy effects and resulting habituation and maximize amygdala reactivity throughout the paradigm. In the control blocks, each of the six shape trios is presented for 4 s with a fixed ISI of 2 s for a total block length of 36 s. Total task time is 390 s.

#### 1.3.5 Latent variable analysis of amygdala activation

The activation variables were represented in terms of lateralization (left vs. right) and also region (basolateral vs. central-medial). Confirmatory factor analysis (CFA) was conducted to determine the best fitting model for the amygdala activation variables. Two CFA models were tested (i.e., left–right vs. BL–CM). Previous meta-analysis suggests the left–right laterality model should produce a better fit (Sergerie et al., 2008). Consistent with the meta-analytic findings, CFA revealed the amygdala activation latent variables (LVs) were best represented in terms of left vs. right activation (CFI = .99; RMSEA = .04), compared to LVs that reflected regional (BL vs. CM) amygdala factors (CFI = .75, RMSEA = .29). Lastly, model analyses were conducted to check the reliability of the amygdala activation variables since this has recently come to the attention of imaging researchers (Elliot et al., 2019). There were 40 participants who completed the activation task at 2 time points, with activation trails separated into A and B blocks. To increase power, a within-subject approach was used for modeling A and B trials with the Mplus complex estimation procedure (Muthén & Muthén, 2013), which brought the total N to 80 (i.e., S1 at T1, S1 at T2, etc.). Thus, this model estimated the reliability across left and right amygdala activation for A and B trials. The model fit better than a region model (BIC: 161 vs. 175), reproduced the data (SRMR = .06), and A and B latent activation factors were strongly correlated (*r*s = .73–.76). Consistent with previous DNS research (Nikolova et al., 2016), males showed slightly higher activation than females, though the effects sizes were quite small (*η* ^*2*^ range = .01 [anger left] to .02 [fear left]).

### 1.4 Data analytic overview

Structural Equation Modeling (SEM) with robust maximum likelihood estimation was used to examine the associations between latent amygdala activation variables and Big Five, INT, and EXT scale scores. SEM is a rigorous statistical method that allows investigators to model the underlying latent variables (LVs) among a set of measures (e.g., amygdala activation) while also allowing the regression of relevant factors or scales (e.g., NEO, INT, EXT) onto the LVs (Walsh et al., 2019). SEM’s advantages over classical test theory include modeling error separately from common variance, specifying clear item-to-factor relations, and yielding robust evidence of construct validity (Strauss & Smith, 2009). A two-index strategy was adopted to assess model fit (Hu & Bentler, 1999), by means of the incremental Comparative Fit Index (CFI) and the absolute Root Mean Square Error of Approximation (RMSEA) index. To avoid falsely rejecting viable latent variable models, the traditional CFI > .90 and RMSEA < .08 cut-offs were used as indicative of acceptable fit because model complexity can increase the difficulty of achieving more conservative levels of fit (Marsh, Hau, & Wen, 2004; West, Taylor, & Wu, 2012). Latent variable analyses were conducted with Mplus (Muthén & Muthén, 2013).

The SEM of primary interest (Model A) was specified so that the amygdala activation LVs predicted both the manifest trait and symptom variables separately, and also the trait domains were specified to predict the symptom variables. As such, the SEM was specified such that there was a linear flow of associations from neurobiology to personality to symptoms (i.e., Amygdala Activation → NEO → INT, EXT) as well as direct associations between activation and symptoms (Amygdala → INT, EXT). As a check on the verisimilitude of this SEM, an alternative model (Model B) was also tested whereby the NEO domains were integrated into either a broad latent INT or latent EXT variable. Specifically, one could suggest that NEO N and (low) E along with the INT manifest variable are indicators of a broad *Internalizing* LV, and that (high) E and (low) A, and (low) C along with the EXT manifest variable are indicators of a broad *Externalizing* LV. Based on previous research (e.g., DeYoung et al., 2008; South & Krueger, 2011), Model B was not expected to fit as well as Model A, which represented the personality and psychopathology domains as separate though significantly interconnected.

To assure that there was invariance of measurement across the sexes, a strong invariance approach was used for the SEM (Walsh et al., 2019). Note, however, that the same substantive results were obtained when the SEMs were run separately for males and females. Indirect effects for the SEMs were also estimated (i.e., activation through personality to psychopathology). All results in the *p* < .01 –.001 range were primarily considered for interpretation, to ensure some level of robustness, though results at the *p* < .05 level are also reported for completeness. Also, 90% confidence intervals were included for all parameters.

## 2. Results

### 2.1 Multiple group SEM results

**Figure 1.**
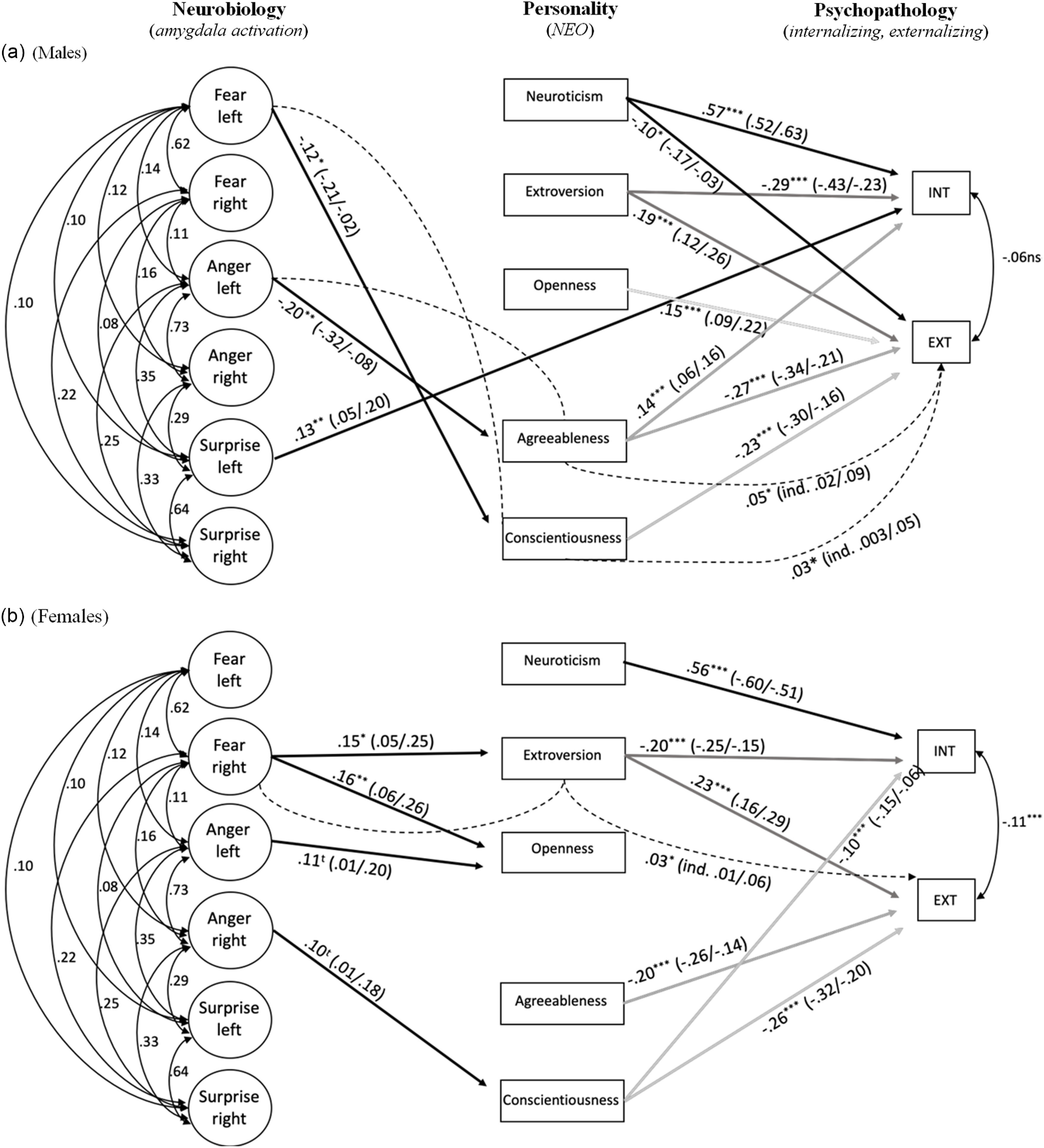
Structural equation model linking neurobiology, personality, and psychopathology.

Note. * = *p* < .05; ** = *p* < .01; *** = *p* < .001; 90% CIs in parentheses. Dashed lines represent indirect (ind.) effects. Big Five correlations for females and males, respectively, −.09 (O & C) to −.48 (O & N); −.07 (O & C) to −.42 (O & N).

For the primary model (Model A), the multiple-group SEM resulted in an excellent fit (CFI = .99; RMSEA = .03), and the loadings for the activation variables were all strong and significant (*p*s < .001).^1^ The majority of the significant SEM parameters were in the *p* < .01–.001 range. For Model B, fit was good (CFI = .94; RMSEA = .07), but Model A fit the data better (BIC_adj_: 76917 vs. 77296). Figure 1 displays the SEM results for males (Panel A) and females (Panel B) separately for Model A.^2^

In terms of associations between amygdala activation and personality, many of the expectations held up, though were moderated by sex. For the males, amygdala activation was inversely associated with Agreeableness (*p* < .01) and Conscientiousness (*p* < .05). In addition, the activation LVs had positive indirect associations with EXT, through personality; however, these only reached *p* < .05. Although not predicted, the surprise activation LV was significantly positively associated with INT for males (*p* < .01). Notably, only the left activation LVs were associated with significant associations for the males.

For the females, the right fear amygdala activation LV was positively associated with Openness (*p* < .01) and Extroversion (*p* < .05). This same LV had a positive indirect association with EXT through Extroversion (*p* < .05). There were two trend positive associations for the anger LVs with Openness and Conscientiousness with parameter 90% CIs outside of zero.

The personality trait domains were linked with INT and EXT in a largely similar fashion across the sexes (*p*s < .01–.001), and the associations were consistent with previous research (Kotov et al., 2011). Neuroticism was positively and Extroversion negatively associated with INT for both males and females. Extroversion was positively associated with EXT, with a slightly larger effect for females. In addition, Agreeableness and Conscientiousness were both negatively associated with EXT across sexes.

There were some interesting differences between the sexes in terms of personality–psychopathology symptom links. For males, Openness was positively associated with EXT and Agreeableness positively associated with INT. Lastly, Neuroticism was negatively associated with EXT for males. Finally, for females, Conscientiousness was positively associated with INT.

## 3. Discussion

The current study used SEM with a large sample to optimize the chances of uncovering NPP links (Abram & DeYoung, 2017). Overall, the results indicated that amygdala activation had direct associations with personality trait expression, and indirect associations (through personality) with symptoms of psychopathology. Despite different levels of analysis involved in uncovering these NPP links (neurobiological, self-report, clinical interview), most of the results had significance levels in the .01–.001 range, and thus may be robust. While the associations uncovered represent small effect sizes they are comparable to other trait/functional brain activity research (Toschi et al., 2018). Tying together different levels of analysis is certainly not easy, and a number of studies have not found significant associations (Gray et al., 2018), but, nonetheless, SEM with large samples may be advantageous, given it has been successfully employed for modeling associations between personality and a range of different domains such as brain structure (Baskin-Sommers et al., 2016), activation (Carré et al., 2013), BIS/BAS dysregulation (Hoppenbrouwers, Neumann et al., 2015), violent criminal acts (Kristic et al., 2018), and emotion dysregulation (Garofalo et al., 2020). The use of a robust modeling approach is not only advantageous for linking different levels of analysis and types of measurement, but also allows investigators to statistically represent a system of variable relations, which is optimal for further research on cybernetic aspects of personality.

Overall, the current results have some correspondence with the cybernetic theories offered by DeYoung (2015) and DeYoung and Krueger (2018a, 2018b). For instance, these investigators proposed that a threat system is linked with one of the stability traits (Neuroticism), which is associated with withdrawal behavior. While the current results did not reveal a link between amygdala activation and Neuroticism, they did show that the fear and anger amygdala activation LVs were associated with a decrease in the other two other stability traits, Agreeableness and Conscientiousness (for males), and activation was positively (indirectly) associated with EXT. Moreover, lower Agreeableness and Conscientiousness were associated with increased in EXT. With respect to Affective Neuroscience Theory, Panksepp and colleagues (Davis & Panksepp, 2011) developed the Affective Neuroscience Personality Scales (ANPS), which were intended to represent neurobiologically based temperament dispositions. It is noteworthy that Panksepp’s ANPS temperament scale taping Anger is strongly inversely associated with Agreeableness (Montag & Davis, 2018). Taken together, the current results suggest that (low) Agreeableness and (low) Conscientiousness, may be linked to the threat system.

Furthermore, DeYoung and Krueger (2018b) indicated that the general factor of psychopathology (*p*) is selectively linked with all three stability traits. In this context, the association between amygdala activation and low Agreeableness and Conscientiousness could be seen as a withdrawal from prosocial propensities. More specifically, decreased Agreeableness and Conscientiousness are well-established correlates of increased EXT (Ruiz et al., 2008), and it may be that movement away from prosocial propensities, goals, and motivations reflects, in part, activation of the threat system. One can view EXT and other forms of aggressivity in terms of the presence of a negative (anti-sociality), but it is also possible to view such behavior in terms of the absence of a positive, such as pro-sociality (Neumann et al., 2007) and affiliative tendencies (Viding & McCrory, 2019). Thus, activation of threat may reduce prosocial affiliative motivations.

The current study had additional results that bear on the cybernetic theories. DeYoung and Krueger (2018a) highlighted that the reward system (e.g., dopamine) is linked to Extroversion, one of the plasticity traits, which activates neurobiological systems (Canli et al., 2002; Cunningham & Kirkland, 2014). The current results suggested that, for females, amygdala activation was linked with Extroversion which was associated with increased EXT. There was also an indirect association of amygdala activation through Extroversion to EXT. Further, it is notable that the dopamine system is also linked to EXT (DeYoung et al., 2006), and the amygdala shows functional connectivity with the ventral striatum (Ousdal et al., 2012), a component of the reward system. As it turns out, the ANPS Seeking subscale, thought to be linked to the ventral striatum, is significantly associated with Extroversion (Montag & Davis, 2018).

Overall, the results highlight that amygdala activation was associated with both stability and plasticity traits. In addition, for both males and females, these meta-trait domains, respectively, were negatively and positively associated with EXT, consistent with previous research (DeYoung et al., 2008). However, that the association between amygdala activation and personality was moderated by sex, but that the associations between personality and psychopathology symptoms (in this case EXT) were similar across sex raises some intriguing issues. For males, amygdala activation was linked to decreases in stability traits, and for females, activation was linked primarily to increases in plasticity traits. Thus, the current results suggest that, for males, decreases in stability traits with amygdala activation may be primary factors for increased EXT. In contrast, for females, the results suggest that increases in plasticity traits (Extroversion) with amygdala activation may be primary factors for increased EXT. This pattern of findings is in accordance with behavior genetic research that found Extroversion had a common genetic basis with EXT for women, and that aspects of low Conscientiousness had a common genetic basis with EXT for men (Kendler & Myers, 2014). As such, sex differences in the expression of the meta-traits, and their association with neurobiology and psychopathology may be a worthwhile avenue to pursue in future research.

Amygdala activity is involved in a host of human (Cunningham & Brosch, 2012; Pessoa, 2010) and animal (Rilling et al., 2012) propensities that have direct relevance to personality trait expression and behavior (Pessoa & Adolphs, 2010). While the current study only employed cross-sectional data, it is reasonable to suggest that there may be reciprocal relations between amygdala activity and personality trait expression. Since amygdala down-regulation is linked with adaptive emotion regulation strategies (Drabant et al., 2009; Gresham & Gullone, 2012; Monk et al., 2008; Morawetz et al., 2017), the “up-regulation” of Agreeableness, and perhaps Conscientiousness, may play a role in such affect regulation propensities (Haas et al., 2007), to help meet the demands of the environment and continue to pursue affiliative goals. Similarly, research has demonstrated that individuals can down-regulate neurobiological responses to affective stimuli, and this ability is linked to greater left prefrontal activation (Davidson et al., 2000). DeYoung (2015) has discussed that Conscientiousness may be linked with greater prefrontal control. Also, higher EXT is linked with lower cognitive skills associated with prefrontal control (DeYoung et al., 2008). In line with these previous findings, the current results indicated decreased amygdala activation (down-regulation) was associated with increased Conscientiousness and lower EXT, consistent with the suggestion that this personality trait may also play a role in affect and behavior regulation.

Amygdala activation with positive affect (Cunningham & Kirkland, 2014) and Extroversion (Canli et al., 2002) have been previously reported, and in line with this research, the current results linked amygdala activation with Extroversion for females. However, the findings of a connection between amygdala activation, Extroversion, and EXT in females are curious. One consideration is that this pattern of associations might represent what DeYoung and Krueger (2018b) have referred to as cybernetic movement toward the “edge of chaos” such that the increased EXT for the females represents an adaptive ‘recklessness’ or ‘disinhibition’ not likely to lead to major psychopathology but rather an expression of cybernetic plasticity.

While the current findings may have some value in furthering our understanding of NPP links, they are at best a small contribution to personality neuroscience research. As Yankori (2015) wrote, it is “unlikely that any single pathway or biological variable will contribute more than a small fraction of the variance …” for understanding personality traits (p. 57). Still, the current results may have at least some generative value for understanding the nature of personality, particularly in terms of neurobiology. Thinking of personality as a motivational goal-oriented cybernetic system is consistent with recent research that finds the Big Five personality domains are replicable predictors of major life outcomes (Soto, 2019). Other research indicates that personality traits are moderately heritable, though heritability decreases with age, and change in personality trait expression is also due to engagement in social roles and life experiences, as well as biological maturation (Kandler, 2012). Personality change appears to be a developmental process, referred to as the maturity principle (Bleidorn, 2015). From a CB5T perspective, personality trait change could involve modifications of the underlying neurobiological processes which inform a given trait propensity. For example, mindfulness practices have been shown to alter neurobiological systems involved in emotion regulation (Hölzel et al., 2013) and are also related to increases in emotional stability and the two plasticity traits (van den Hurk et al., 2011). Taken together, these studies and the current results highlight the adaptive (or maladaptive) nature of personality, supported by neurobiological systems. Moreover, neurobiology could be influenced, in a reciprocal fashion, by increasing the expression of personality traits linked to affiliation (Bickart et al., 2012; Klimecki et al. 2013), or mindfulness (Hölzel et al., 2011; Taren et al., 2013) which may then lead to better emotion regulation as well as changes in structural and functional characteristics of the amygdala.

### 3.1 Caveats

Despite the use of a large sample and sophisticated modeling, the current study is not without its limitations. The data were cross-sectional and thus the modeling results should not be interpreted with respect to strict causality. Second, while an attempt was made to derive directional hypotheses, there were nevertheless a number of statistical analyses, and thus some of the findings could be due to chance. Lastly, in the current study, only amygdala activation was incorporated to examine neurobiology–personality associations, and, therefore, was not able to address the importance of other brain regions, and the connections between regions, which no doubt also play a role in how personality propensities are informed by neurobiological factors (DeYoung & Gray, 2009).

## 4. Conclusion

The current study found evidence for meaningful links between neurobiology, personality trait expression, and symptoms of psychopathology. Most of these associations were of small magnitude, though still appeared to be of a relatively robust nature, in part due to the reliance on substantial sample size (Allen & DeYoung, 2017). Moreover, the results were in line with previous cybernetic and affective neuroscience theories, which adds to the existing support for these theories, and perhaps help to provide a deeper understanding of personality neuroscience.

## Footnotes

1 A strong invariance multiple-group confirmatory factor analysis (CFA) was also run to check on the measurement model and pattern of variable correlations. As it turns out, the MG-CFA had identical fit with the MG-SEM, and thus represents an alternative equivalent model (MacCallum et al., 1993). As such, the pattern of variable correlations matched the pattern of SEM parameters.

2 Model A was also run with the total sample (CFI = .99, RMSEA = .03), with many similarities and some differences between male/female models. Most notable difference was attenuated SEM parameters.
